# A connecter-like factor, CacA, links RssB/RpoS and the CpxR/CpxA two-component system in *Salmonella*

**DOI:** 10.1186/1471-2180-12-224

**Published:** 2012-10-02

**Authors:** Akinori Kato, Hironori Hayashi, Wataru Nomura, Haruka Emori, Kei Hagihara, Ryutaro Utsumi

**Affiliations:** 1 Department of Advanced Bioscience, Graduate School of Agriculture, Kinki University, Nakamachi, Nara 631–8505, 3327-204, Japan

**Keywords:** Two-component system, Connector, Network, RssB, RpoS, CacA, CpxR/CpxA

## Abstract

**Background:**

Bacteria integrate numerous environmental stimuli when generating cellular responses. Increasing numbers of examples describe how one two-component system (TCS) responds to signals detected by the sensor of another TCS. However, the molecular mechanisms underlying this phenomenon remain poorly defined.

**Results:**

Here, we report a connector-like factor that affects the activity of the CpxR/CpxA two-component system in *Salmonella enterica* serovar Typhimurium. We isolated a clone that induced the expression of a *cpxP-lac* gene fusion from a high-copy-number plasmid pool of random *Salmonella* genomic fragments. A 63-amino acid protein, CacA, was responsible for the CpxA/CpxR-dependent activation of the *cpxP* gene. The CpxR-activated genes *cpxP* and *spy* exhibited approximately 30% and 50% reductions in transcription, respectively, in a clean *cacA* deletion mutant strain in comparison to wild-type. From 33 response regulator (RR) deletion mutants, we identified that the RssB regulator represses *cacA* transcription. Substitution mutations in a conserved -10 region harboring the RNA polymerase recognition sequence, which is well conserved with a known RpoS -10 region consensus sequence, rendered the *cacA* promoter RpoS-independent. The CacA-mediated induction of *cpxP* transcription was affected in a *trxA* deletion mutant, which encodes thioredoxin 1, suggesting a role for cysteine thiol-disulfide exchange(s) in CacA-dependent Cpx activation.

**Conclusions:**

We identified CacA as an activator of the CpxR/CpxA system in the plasmid clone. We propose that CacA may integrate the regulatory status of RssB/RpoS into the CpxR/CpxA system. Future investigations are necessary to thoroughly elucidate how CacA activates the CpxR/CpxA system.

## Background

The two-component system (TCS) is one of the most ubiquitous signal transduction systems in bacteria [1]. A prototypical TCS harbors a sensor histidine kinase (HK), which is often integrated into the inner membrane, and a response regulator (RR), which is predominantly a cytoplasmic DNA-binding transcription factor. In the presence of a specific activating signal, the sensor HK is autophosphorylated, and a phosphoryl group is subsequently transferred to a conserved aspartate residue in its cognate RR, thus changing gene expression patterns and cell physiology. Each TCS responds to specific environmental signals but elude identification even in the well-investigated organisms *Escherichia coli* and *Salmonella*. Due to the high levels of sequence and structure similarity among different TCSs, cross-talk (i.e., phosphotransfer from a HK to its non-cognate RR) may occur in at least some circumstances. However, cross-talk is extremely rare due to the kinetic preference of a sensor HK for its cognate RR [2] and their phosphatase activities [3].

To date, several small proteins connecting TCSs have been reported in *Salmonella* and *E. coli*[4,5]. For example, the 85-amino acid PmrD protein, which is transcriptionally induced by the PhoP/PhoQ system under low Mg^2+^ conditions, binds to the phosphorylated form of the regulator PmrA and hinders its dephosphorylation by the cognate sensor PmrB [6]. Therefore, expression of PmrA-activated genes, some of which are responsible for polymixin B resistance and iron resistance in *Salmonella,* is induced even in the absence of an Fe^3+^ signal [7]. The small anti-adapter proteins IraP and IraM, which promote the stability of the stationary phase sigma S factor (RpoS) of RNA polymerase by hindering an RR (RssB), are also transcriptionally activated by the PhoP/PhoQ system in response to low Mg^2+^ conditions in *Salmonella*[8] and *E. coli*[9], respectively. In contrast to these cytosolic connectors, the small inner membrane proteins SafA (B1500) [10] and MzrA [11] were identified as signal transducers between two TCSs by targeting downstream sensor HKs. SafA elicits a response from the PhoQ sensor to the PhoP regulator even under high Mg^2+^ conditions when the EvgS1 mutan protein [12] induces the EvgA-activated *safA* gene constitutively [10]. Alternatively, MzrA interacts with the EnvZ sensor to control OmpR-regulated gene transcription when *mzrA* expression is induced in a constitutively activated CpxA* mutant background [13] in *E. coli*. The membrane peptide MgrB [14,15], which corresponds to a single TCS, communicates the activation status of the PhoP regulator to its cognate sensor PhoQ in *E. coli* and *Salmonella*[15]. In contrast, the unique membrane peptide PmrR mediates the feedback control of the PmrA/PmrB system indirectly in *Salmonella*[16].

The CpxR/CpxA system regulates pilus assembly, adherence, and biofilm development in response to envelope stress and is required for host cell invasion in several species, including pathogenic *E. coli* and *Salmonella*[17]. The periplasmic chaperone CpxP binds to both the CpxA periplasmic domain and to certain misfolded proteins, which are degraded by the periplasmic protease DegP, therefore integrating information about their turnover status to the kinase activity of CpxA [18-20]. The outer membrane lipoprotein NlpE activates the CpxA protein upon its overexpression [21] and is required for CpxA protein activation after adhering to hydrophobic surfaces [22]. Additional upstream components have been proposed to integrate other stresses in a process that is independent of the CpxP and NlpE pathways [17,23]. For example, the CpxR/CpxA system confers a copper resistance phenotype even in CpxP and NlpE mutants [24]. Notably, *nlpE* (*cutF* or STM0241) is a pseudogene in *Salmonella*[25].

Here, we aimed to identify candidate connector genes that may integrate the signals of other systems. We identified a small protein as a novel connector-like factor from screening high copy plasmid clones that could affect the CpxR/CpxA system status.

## Results

### Identification of a plasmid clone that activates *cpxP* transcription

**Figure 1:**
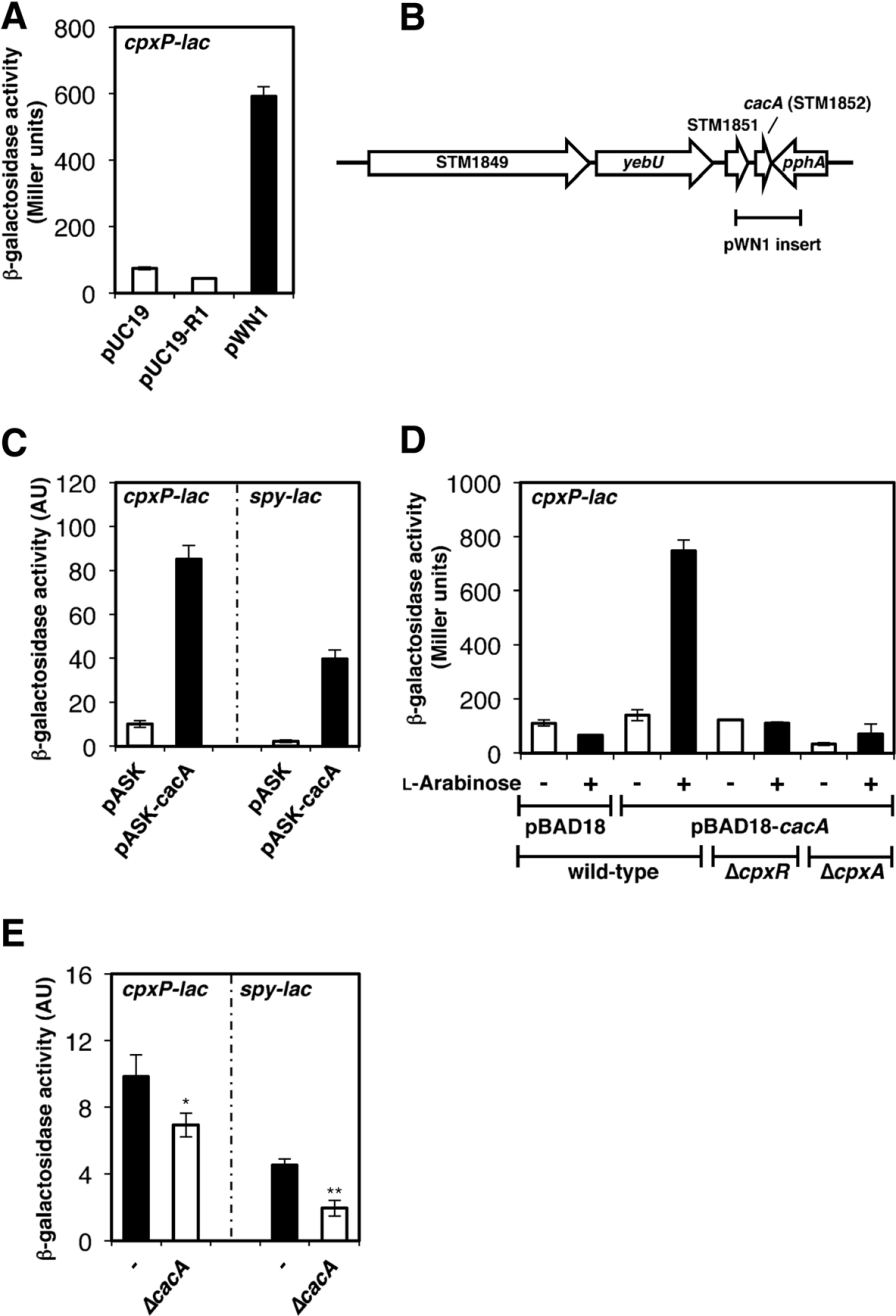
**The identification of a novel connector-like factor, CacA. A.** β-galactosidase activity from a *cpxP-lac* transcriptional fusion expressed in the wild-type strain (AK1052) harboring pUC19, pUC19-R1, and pWN1. Bacteria were grown for 4 h in LB before β-galactosidase activity was measured (Miller units). The data correspond to the means of two independent experiments performed in duplicate, and the error bars represent standard deviations. **B.** A genetic map of the *cacA* (STM1852) locus in *Salmonella*. Each arrow indicates a gene and its orientation in the chromosome. The chromosomal location corresponding to the inserted DNA fragment of the pWN1 plasmid clone is indicated by a horizontal bar. **C.** β-galactosidase activity from *cpxP-lac* or *spy-lac* transcriptional fusions in a wild-type (AK1052 or AK1053) strain harboring pASK or pASK-cacA. Bacteria were grown for 2 h in LB in the presence of 0.2 μg/ml anhydrotetracycline (ATc) before β-galactosidase activity was measured (arbitrary units) as described [42]. The data correspond to the means of three independent experiments performed in duplicate, and the error bars represent standard deviations. **D.** β-galactosidase activity from a *cpxP-lac* transcriptional fusion in the wild-type strain (AK1052) harboring pBAD18 or pBAD18-cacA and the Δ*cpxR* mutant (AK1061) and Δ*cpxA* mutant (AK1062) strains harboring pBAD18-cacA. Bacteria were grown for 4 h in LB in the presence (+) or absence (−) of 5 mM L-arabinose before β-galactosidase activity was measured (Miller units). The data correspond to the means of two independent experiments performed in duplicate, and the error bars representstandardrepresent standard deviations. **E.** β-galactosidase activity from *cpxP-lac* or *spy-lac* transcriptional fusions in a wild-type strain (−; AK1052 or AK1053) and a Δ*cacA* mutant strain (AK1075 or AK1076). Bacteria were grown for 4 h in N-minimal medium, pH 7.7 with 10 μM Mg2+ before β-galactosidase activity was measured (arbitrary units) as described [42]. The data correspond to the means of three independent experiments performed in duplicate, and the error bars represent standard deviations. Single and double asterisks indicate p < 0.05 and p < 0.01, respectively, using an unpaired t test for analysis.

To conduct a genetic screen for novel connector proteins acting on the CpxR/CpxA system, we constructed a strain harboring a *cpxPlac* transcriptional fusion in *Salmonella*. The *cpxP* gene was chosen as a readout of the activation status of the CpxR/CpxA system because it is likely directly regulated exclusively by this system, unlike other CpxR-activated genes that are also controlled by envelope stress-responsive systems [26-28]. The *lacZY* genes were inserted after the *cpxP* stop codon to ensure that the CpxP protein retained the ability to repress the CpxR/CpxA system. Then, *Salmonella* chromosomal DNA was partially digested with *Sau*3AI and ligated with the high-copy-number plasmid pUC19 (digested with *Bam*HI) to generate a DNA fragment library. Of approximately 10,000 *cpxP-lac Salmonella* transformants, a plasmid clone termed pWN1 yielded stable blue colonies on LB plates containing 5-bromo-4-chloro-3-indolyl-β-D-galactoside (X-gal) and ampicillin and was isolated four times. The blue color of the pWN1 strain was due to elevated *cpxP-lac* fusion expression. We demonstrated that this strain exhibit ~8-fold higher β-galactosidase activity than the same strain harboring the vector control or the plasmid clone pUC19-R1 that was randomly selected during the screening as a negative control (Figure 1A). Sequence analysis revealed that pWN1 harbors only the intact STM1852 open reading frame (ORF), which appeared to encode a 63-amino acid protein with no homology to any protein of known function, as well as the 3’ region of STM1851 and the 5’ region of *pphA* (Figure 1B). Expression of STM1852 from tetracycline- (Figure 1C) or L-arabinose- (Figure 1D) inducible promoters recapitulated the increase in the β-galactosidase activity observed in the *cpxPlac* strain, supporting the hypothesis that STM1852 was affecting *cpxP* transcription in the pWN1 plasmid. Here, we named STM1852 “Cpx activating connector-like factor A”, or CacA.

### CacA-mediated *cpxP* activation is dependent on the CpxR/CpxA system

The results described above demonstrated that *cpxP* transcription was induced when CacA was expressed from a high-copy-number plasmid or from a heterologous promoter in an inducer-dependent manner. Next, we compared the β-galactosidase activities of the *cpxP-lac* fusion from *cpxR* and *cpxA* mutant strains harboring pBAD18*-cacA* to an isogenic *cpxR*^*+*^*A*^*+*^ strain containing the same plasmid (Figure 1D). We determined that CacA acts upstream of the CpxR/CpxA system because the activities of the *cpxR* and *cpxA* mutant strains expressing CacA were comparable to that of the isogenic *cpxR*^*+*^*A*^*+*^ strain with vector (i.e., pBAD18) alone (Figure 1D). This was further supported by the observation that another CpxR-activated gene, *spy,* was induced by CacA protein overexpression (Figure 1C). Moreover, CacA likely acts on the CpxR/CpxA system specifically because expression of CacA did not affect genes under the direct control of other TCSs (data not shown).

### *cacA* transcription is activated by RpoS but repressed by RssB

**Figure 2:**
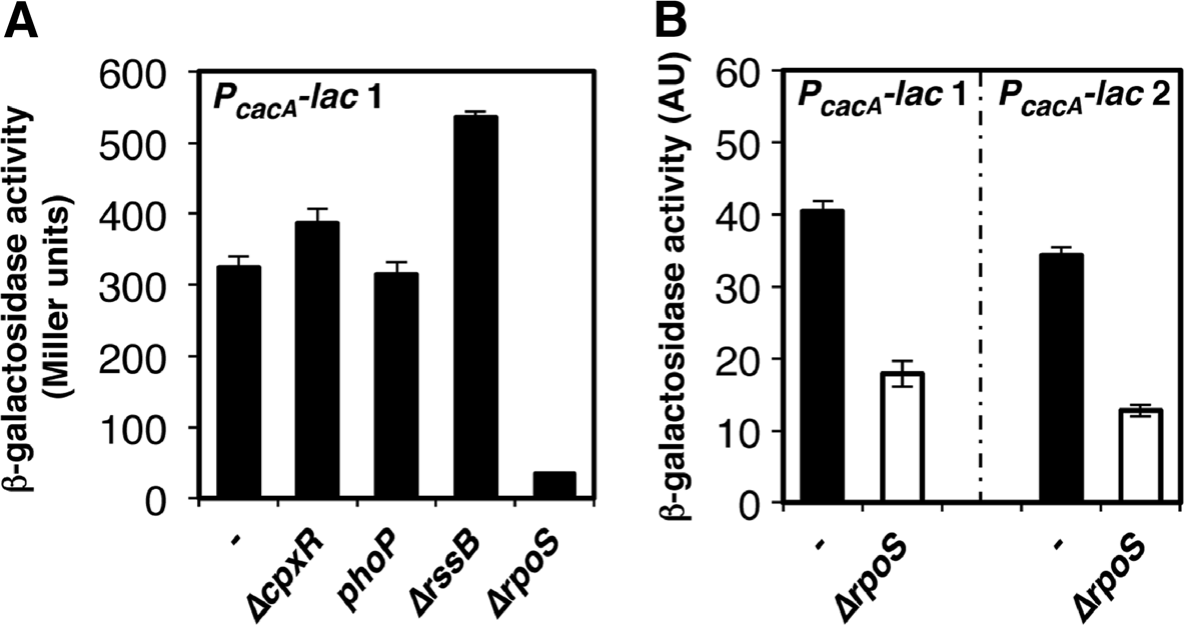
**Transcription of the cacA gene is activated by RpoS but repressed by RssB. A. **β-galactosidase activity from a *PcacA-lac* transcriptional fusion 1 in the wild-type (−; AK1056), Δ*cpxR *mutant (AK1063), *phoP* mutant (AK1064), Δ*rssB* mutant (AK1065), and Δ*rpoS* mutant (AK1066) strains. Bacteria were grown for 4 h in LB before β-galactosidase activity was measured (Miller units). The data correspond to the means of two independent experiments performed in duplicate, and the error bars represent standard deviations. **B.** β-galactosidase activity from *PcacA-lac* transcriptional fusion 1 or 2 in a wild-type strain (−; AK1056 or AK1067) and a Δ*rpoS* mutant strain (AK1059 or AK1071). Note that the *PcacA-lac* 1 strain contains a DNA fragment encompassing the 3’ region (80 bp) of STM1851 and the intergenic region (110 bp) between STM1851 and *cacA*, whereas the *PcacA-lac* 2 strain harbors only the intergenic region (110 bp) between STM1851 and *cacA* preceding the *lacZ* gene (See **Methods**). Bacteria were grown for 4 h in LB before β-galactosidase activity was measured (arbitrary units) as described [42]. The data correspond to the means of three independent experiments performed in duplicate, and the error bars represent standard deviations. The data in the panels **A** and **B** were obtained using two different methods.

**Figure 3:**
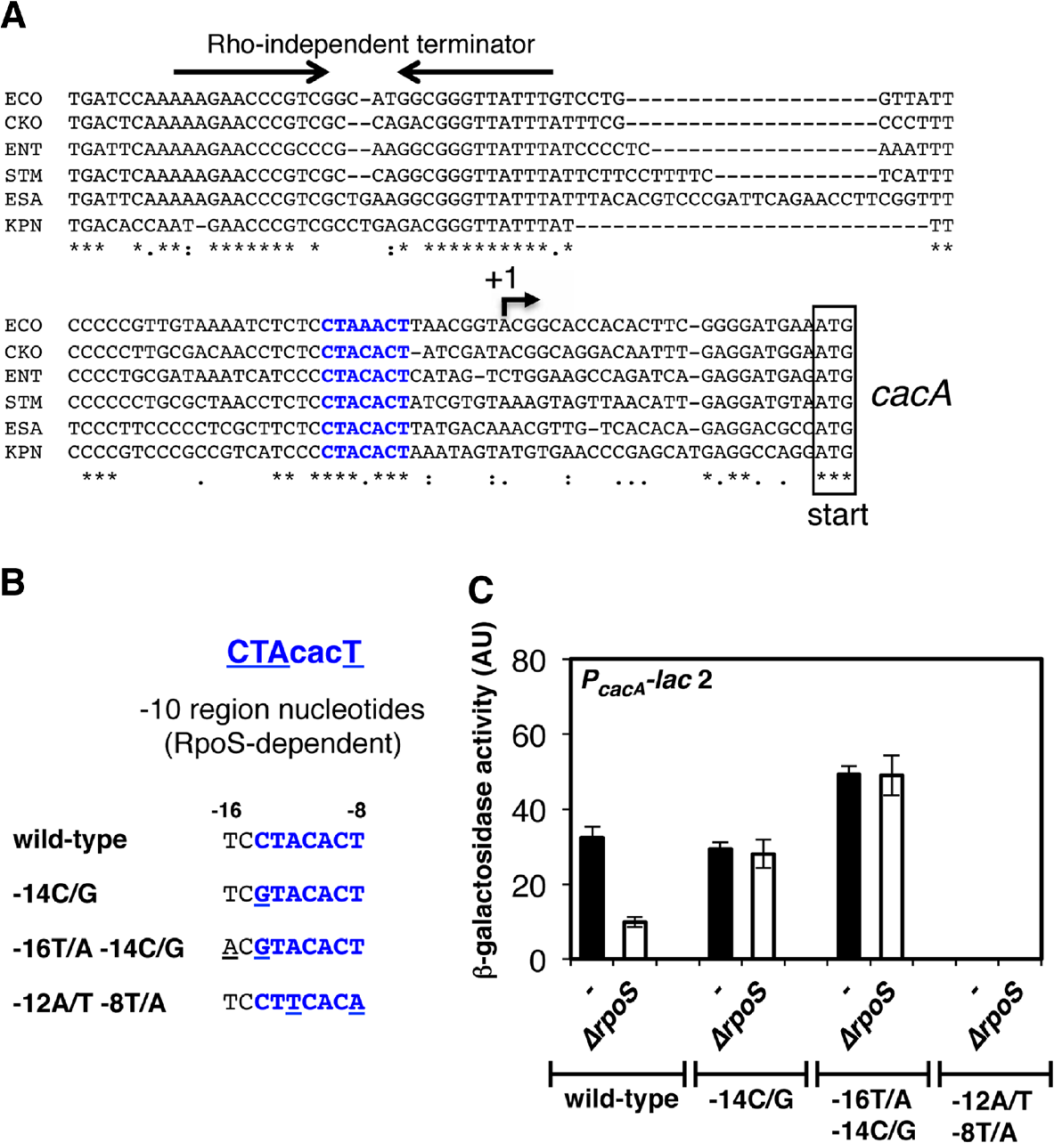
**The *cacA *promoter harbors a conserved -10 region sequence that is crucial for RpoS-dependent regulation. A**. Alignment of the DNA sequences of the intergenic region between the *cacA*-coding region and its upstream ORF (STM1851) in *E. coli* (ECO), *C. koseri* (CKO), *Enterobacter* sp. 638 (ENT), *S. enterica* serovar Typhimurium LT2 (STM), *Klebsiella pneumoniae* (KPN)*,* and *C. sakazakii* (ESA)*.* Asterisks correspond to nucleotides that are conserved in all listed species. Twin dots and single dots indicate conservative and semiconservative substitutions, respectively. The -10 region sequence is marked in bold blue letters. The bent arrow indicates the transcription start site (TSS) of the *cacA* transcript, as determined by a recent report [30] (designated position +1). The inverted arrows indicate predicted Rho-independent terminator sequences. The initiation codons for the *cacA* gene are boxed. **B**. Designated mutations in the *cacA* promoter. The -10 region sequence (**CTAcacT** from -13 to -7) [29] represents a consensus sequence that is recognized by RpoS. The -10 region sequence of the *cacA* promoter is highlighted in blue. The numbers shown above the wild-type sequence are the positions relative to the *cacA* TSS [30]. The substituted nucleotides (-14C/G, -16T/A -14C/G, and -12A/T -8T/A) are underlined. **C**. β-galactosidase activity from a P_*cacA*_*-lac* transcriptional fusion 2 in the wild-type (−; AK1067), Δ*rpoS* mutant (AK1071), -14C/G *cacA* promoter mutant (AK1068), Δ*rpoS* -14C/G *cacA* promoter mutant (AK1072), -16T/A -14C/G *cacA* promoter mutant (AK1069), Δ*rpoS* -16T/A-14C/G *cacA* promoter mutant (AK1073), -12A/T -8T/A *cacA* promoter mutant (AK1070), and Δ*rpoS* -12A/T -8T/A *cacA* promoter mutant (AK1074) strains. Bacteria were grown for 4 h in LB before β-galactosidase activity was measured (arbitrary units) as described [42]. The data correspond to the means of three independent experiments performed in duplicate, and the error bars represent standard deviations.

Next, we asked whether the *cacA* gene might be regulated by an undefined upstream TCS. To examine candidate TCSs that could potentially affect *cacA* transcription, we constructed a strain with a *cacA* promoter-*lac* fusion 1 (i.e., P_*cacA*_*-lac* 1) at the *pgtP* locus on the *Salmonella* chromosome. Then, 33 RR mutant stocks were independently transduced into the P_*cacA*_*-lac* 1 strain by phage P22. Whereas most RR mutants exerted minor or no effects on transcription from the *cacA* promoter (data not shown, Figure 2A), the *rssB* mutant exhibited a ~1.5-fold increase in *cacA* promoter activity (Figure 2A). Because RssB is the adaptor protein that recruits RpoS to the ClpXP protease, we examined the effect of a Δ*rpoS* mutant on transcription from the *cacA* promoter. As expected, the *rpoS* gene was required for *cacA* expression (Figures 2A and 2B). Consistent with these observations, an alignment of the *cacA* promoter regions from *Salmonella* and its related enteric species revealed a conserved sequence that is present in an RpoS-dependent consensus -10 region sequence (**CTAcacT** from -13 to -7) [29] (Figure 3A).

Moreover, although the location of the predicted -10 region correlates well with a transcription start site (TSS) determined by a genome-scale precise mapping of TSSs that covered 78% of the *Salmonella* ORFs [30], no obvious typical -35 region sequence exists upstream of the -10 nucleotides (Figure 3A). We mutated this -10 sequence from TC**CTACACT** to TC**GTACACT** (-14C/G), AC**GTACACT** (-16T/A-14C/G), or TC**CTTCACA** (-12A/T -8T/A) and analyzed their effects on *cacA* transcription (Figures 3B and 3C). In the Δ*rpoS* mutant, the β-galactosidase activity of the *cacA* promoter was approximately 1/3 of wild-type levels (Figure 3C). However, the β-galactosidase activities from the *cacA* promoter containing -14C/G or -16T/A -14C/G substitutions were not affected by the Δ*rpoS* mutation after 4 h of growth in LB, indicating that these substitution mutations rendered the *cacA* promoter RpoS-independent (Figure 3C). Conversely, when the essential nucleotides -12A and -8T of the canonical -10 region sequence, which permits recognition by both RpoD and RpoS, were mutated, *cacA* promoter activity was abolished independent of RpoS presence (Figure 3C). Taken together, these results demonstrated that the activation of the *cacA* promoter is dependent on the -10 region sequence, which harbors an RpoS recognition site.

### Transcription of the CpxR-activated genes *cpxP* and *spy* is attenuated in a *cacA* mutant

Because RpoS activates *cacA* expression, we assessed whether a *cacA* deletion mutation would affect transcription of the CpxA/CpxR-dependent *cpxP* and *spy* genes in low Mg^2+^, the conditions under which the PhoQ/PhoP-activated IraP prevents the RssB/ClpXP-mediated degradation of RpoS, even at log phase [8]. We determined that CacA participates in CpxA/CpxR system activation because *cpxP* and *spy* expression levels were reduced by approximately 30% and 50%, respectively, in the *cacA* deletion mutant compared with wild-type (Figure 1E).

### Thioredoxin 1 is required for the CacA-mediated activation of the CpxR/CpxA system

**Figure 4:**
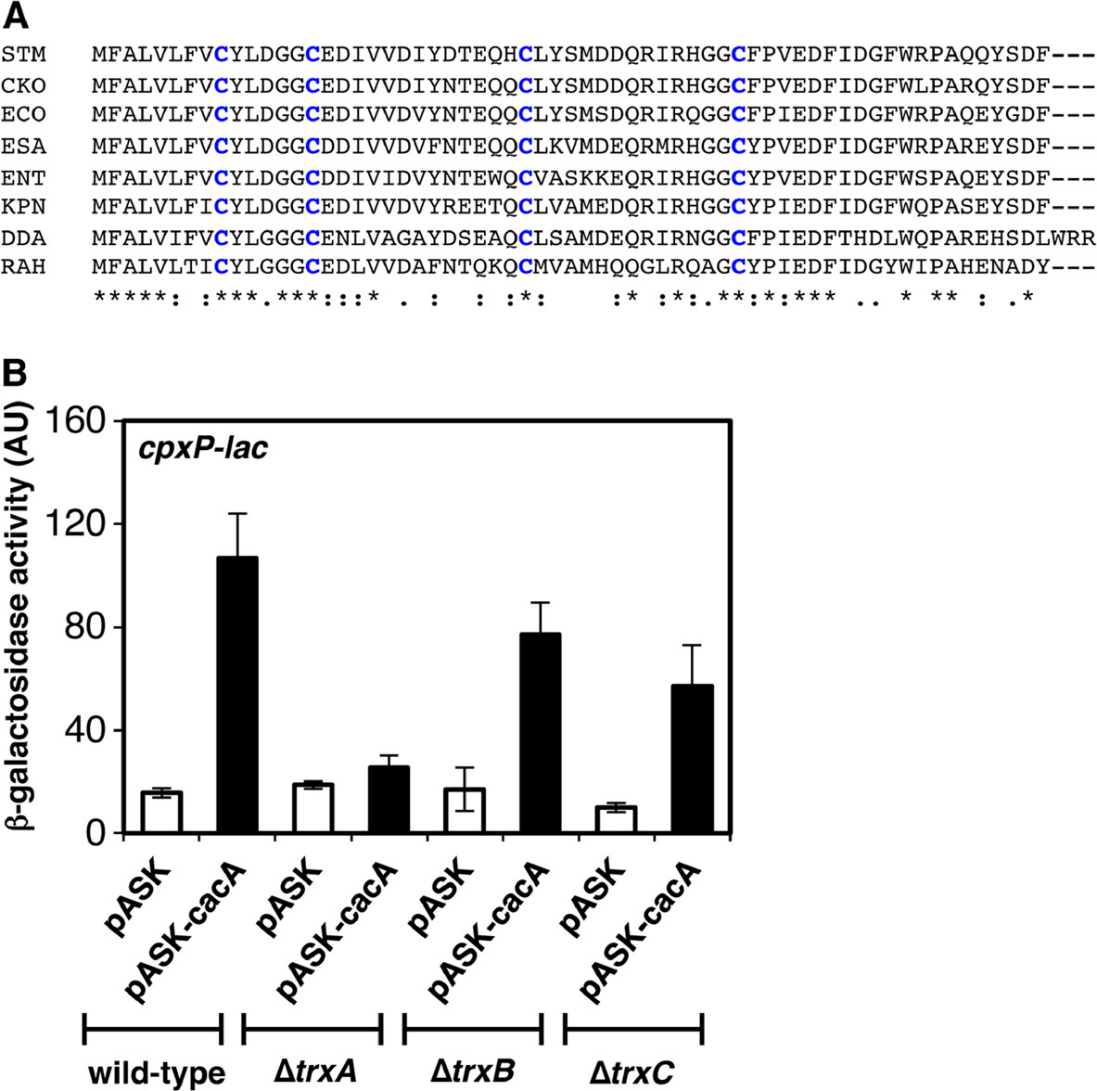
**The CacA-dependent activation of the CpxR/CpxA requires functional thioredoxin 1. A**. Alignment of the amino acid sequences of the CacA protein of *S. enterica* serovar Typhimurium LT2 (STM), *C. koseri* (CKO), *E. coli* (ECO), *C. sakazakii* (ESA), *Enterobacter* sp. 638 (ENT), *Klebsiella pneumoniae* (KPN)*, D. dadantii Ech703* (DDA), and *Rahnella sp. Y9602* (RAH)*.* Conserved cysteine residues are marked in bold blue letters. Asterisks indicate amino acids that are conserved in all listed species. Twin dots and single dots indicate conservative and semiconservative substitutions, respectively. **B**. β-galactosidase activity from a *cpxP-lac* transcriptional fusion in the wild-type (AK1052), Δ*trxA* mutant (AK1080), Δ*trxB* mutant (AK1081), and Δ*trxC* mutant (AK1082) strains harboring plasmids pASK or pASK-*cacA*. Bacteria were grown for 2 h in LB in the presence of 0.2 μg/ml ATc before β-galactosidase activity was measured (arbitrary units) as described [42]. The data correspond to the means of three independent experiments performed in duplicate, and the error bars represent standard deviations.

Pull-down experiment of the Glutathione S Transferase (GST)-CacA fusion protein recovered the GroEL and thioredoxin 1 (TrxA) proteins, suggesting that they interact directly with CacA (data not shown). Because GroEL has been shown to associate with proteins that are overexpressed, we did not investigate its role further. Instead, we focused on the effect of TrxA on the CacA-mediated activation of the CpxR/CpxA system because CacA orthologs contain four conserved cysteine residues (Figure 4A) and because TrxA catalyzes thiol disulfide redox reactions in a variety of substrate proteins [31]. We investigated TrxC, another thioredoxin, and TrxB, which participates in the regeneration of reduced TrxA and TrxC [31], as controls. Whereas mutations in *trxA, trxB*, and *trxC* did not affect *cpxP* transcription in strains harboring vector alone, the *trxA* mutant expressing CacA significantly decreased the levels of *cpxP* transcription compared to wild-type expressing CacA (Figure 4B). These results indicate that TrxA is required for the CacA-mediated activation of the CpxR/CpxA system. This suggests that cysteine thiol-disulfide exchanges participate in CacA-dependent Cpx activation.

## Discussion

**Figure 5:**
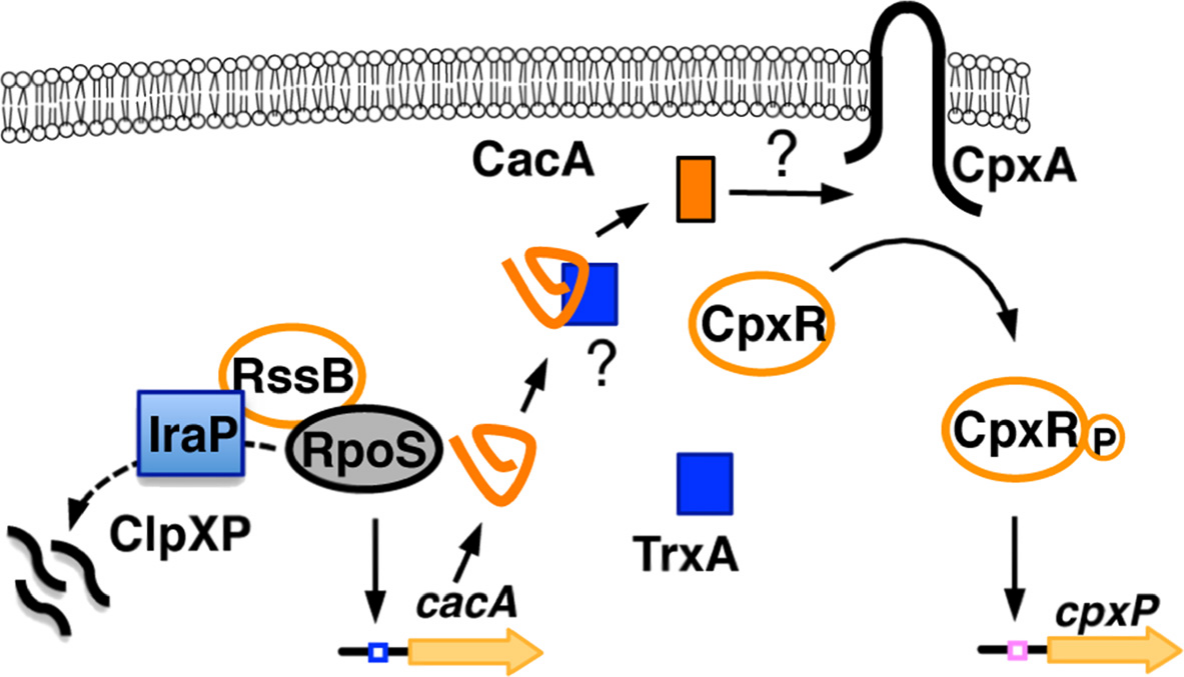
**A model for the regulatory interactions between RssB/RpoS and the CpxR/CpxA system.** RpoS accumulates during stationary phase and log phase, when the small anti-adopter protein IraP inhibits the RssB/ClpXP-mediated degradation of RpoS in low Mg^2+^ conditions [8]. RpoS induces expression of CacA, which stimulates the CpxR/CpxA system thus activating *cpxP* transcription. TrxA functionally associates with CacA-mediated Cpx induction.

We identified CacA, encoded on a plasmid clone, as a novel connector-like factor that activated the CpxR/CpxA system from screening a library of high-copy-number plasmids containing various *Salmonella* chromosomal DNA fragments. CacA appears to exclusively act on the CpxR/CpxA system because a similar induction was not observed in other TCS reporter strains with the same clone. This observation was not just an artifact of CacA overexpression or from its expression driven by a heterologous promoter because deleting this gene revealed a moderate decrease in transcription of the *cpxP* and *spy* genes, which are directly regulated by the CpxR/CpxA system. Moreover, the activation of the *cacA* gene promoter is, at least in part, dependent on RpoS, the stability of which is subject to RssB/ClpXP-mediated processability and the -10 region sequence. Taken together, we hypothesize that CacA may integrate information about the regulatory status of RssB/RpoS into the CpxR/CpxA system (Figure 5). However, future investigations are necessary to fully elucidate the mechanism of CacA-mediated CpxR/CpxA activation.

Several assessments of how the CacA protein activates CpxR-regulated genes were attempted. However, we did not detect a physical association between CacA and the CpxR/CpxA system. For example, no significant interaction was observed between the CacA protein and the CpxR/CpxA system in our bacterial two-hybrid system analyses (data not shown), although we cannot completely dismiss that these proteins do not interact directly. Instead, thioredoxin 1 amino acid sequences were recovered by our pull-down assay. *trxA* inactivation impacted the activation of the CpxR/CpxA system by CacA, which possesses the conserved cysteine residues. This is in contrast to a report that demonstrated that a *dsbD* mutation activated the CpxR/CpxA system in *Vibrio cholerae*[32], where the DsbC-DsbD pathway promotes proper folding of substrate proteins with disulfide bond(s) at the periplasm using the cytoplasmic reducing ability of thioredoxin [33]. Moreover, the cysteine residues of NlpE are critical for activating the CpxR/CpxA system in *E. coli*[34], and a periplasmic LolA derivative with an artificial disulfide bond activates the CpxR/CpxA system [35]. Notably, perturbing the oxidizing environment of the periplasm in *dsbA* and *dsbB* mutants or treating wild-type cells with dithiothreitol (DTT) activated the PhoP/PhoQ system in *E. coli*[36]. Disruption of disulfide bond formation affects this system largely via an additional small protein component, MgrB, and its conserved cysteine residues.

Currently, we cannot exclude the possibility that the interaction between CacA and TrxA is an artifact CacA protein overexpression because TrxA interacts with many proteins, including the RR RcsB [37]*.* Because we were unable to detect the 63-amino acid CacA protein at native levels, we employed a larger tag or carrier protein in several biochemical experiments, including the pull-down assay. Protein instability likely precludes thorough analysis of small proteins of less than 50 amino acids or so [38]. Notably, deletion of *trxA* did not impact *cpxP* transcription levels in normal growth conditions (e.g., LB medium). More strict conditions need to be tested, as some small proteins accumulated within bacterial cells upon exposure to sodium dodecyl sulfate (SDS) and ethylenediaminetetraacetic acid (EDTA) [38].

The specificity that TCS connectors exhibit for their targets is likely a key contributing factor in the fidelity of the integration of TCS signals at a post-translational level. In fact, the PmrD connector protein can inhibit the dephosphorylation of phospho-PmrA but not of its closest homolog, the response regulator YgiX [6]. Although recognizing novel connectors in genomic sequences based on their uniqueness is far from trivial, genetic approaches will continue to help elucidate links amongst TCSs.

## Conclusions

In this study, we identified the CacA protein as an activator of the CpxR/CpxA system. This factor may be another example of an emerging class of small proteins [39] that function as nodes in the TCS network and function to integrate their signaling pathways in *Salmonella*.

## Methods

### Bacterial strains, plasmids, primers, and growth conditions

Bacterial strains and plasmids used in this study are listed in Table 1. Primers used in this study are listed in Table 2. All *S. enterica* serovar Typhimurium strains are derived from wild-type 14028s and were constructed by phage P22-mediated transduction as previously described [40]. Bacteria were grown at 37°C in N-minimal media [41] buffered with 50 mM Bis-Tris, pH 7.7, and supplemented with 0.1% casamino acids, 38 mM glycerol and 10 μM or 10 mM MgCl2. *E. coli* DH5 α was used for preparing plasmid DNA. Ampicillin and kanamycin were used at 50 μg/ml, chloramphenicol at 20 μg/ml and tetracycline at 10 μg/ml.

**Table 1:** Bacterial Strains and Plasmids Used in This Study

**Strain or plasmid**	**Description**	**Reference or source**
***S. enterica***		
14028s	Wild-type	ATCC
MS7953s	*phoP*::Tn*10*	[48]
AK1052	*cpxP-lacZ*^*+*^*Y*^*+*^	This work
AK1053	*spy-lacZ*^*+*^*Y*^*+*^	This work
AK1054	*pgtP-lacZ*^*+*^*Y*^*+*^	This work
AK1055	*pgtP-tetA-lacZ*^*+*^*Y*^*+*^	This work
AK1056	P_*cacA*_-*lacZ*^*+*^*Y*^*+*^1	This work
AK1007	Δ*cpxR*::Cm^R^	[16]
AK1057	Δ*cpxA*::Cm^R^	This work
AK1058	Δ*rssB*::Cm^R^	This work
AK1059	Δ*rpoS*::Cm^R^	This work
AK1060	Δ*cacA*::Cm^R^	This work
AK1061	*cpxP-lacZ*^*+*^*Y*^*+*^1 Δ*cpxR*::Cm^R^	This work
AK1062	*cpxP-lacZ*^*+*^*Y*^*+*^1 Δ*cpxA*::Cm^R^	This work
AK1063	P_*cacA*_*-lacZ*^*+*^*Y*^*+*^1 Δ*cpxR*::Cm^R^	This work
AK1064	P_*cacA*_*-lacZ*^*+*^*Y*^*+*^1 *phoP*::Tn*10*	This work
AK1065	P_*cacA*_*-lacZ*^*+*^*Y*^*+*^1 Δ*rssB*::Cm^R^	This work
AK1066	P_*cacA*_*-lacZ*^*+*^*Y*^*+*^1 Δ*rpoS*::Cm^R^	This work
AK1067	P_*cacA*_*-lacZ*^*+*^*Y*^*+*^2	This work
AK1068	P_*cacA*-14C/G_*-lacZ*^*+*^*Y*^*+*^2	This work
AK1069	P_*cacA*-16T/A-14C/G_*-lacZ*^*+*^*Y*^*+*^2	This work
AK1070	P_*cacA*-12A/T-8T/A_*-lacZ*^*+*^*Y*^*+*^2	This work
AK1071	P_*cacA*_*-lacZ*^*+*^*Y*^*+*^2 Δ*rpoS*::Cm^R^	This work
AK1072	P_*cacA*-14C/G_*-lacZ*^*+*^*Y*^*+*^2 Δ*rpoS*::Cm^R^	This work
AK1073	P_*cacA*-16T/A-14C/G_*-lacZ*^*+*^*Y*^*+*^2 Δ*rpoS*::Cm^R^	This work
AK1074	P_*cacA*-12A/T-8T/A_*-lacZ*^*+*^*Y*^*+*^2 Δ*rpoS*::Cm^R^	This work
AK1075	Δ*cacA cpxP-lacZ*^*+*^*Y*^*+*^	This work
AK1076	Δ*cacA spy-lacZ*^*+*^*Y*^*+*^	This work
AK1077	Δ*trxA*::Cm^R^	This work
AK1078	Δ*trxB*::Cm^R^	This work
AK1079	Δ*trxC*::Cm^R^	This work
AK1080	*cpxP-lacZ*^*+*^*Y*^*+*^Δ*trxA*::Cm^R^	This work
AK1081	*cpxP-lacZ*^*+*^*Y*^*+*^Δ*trxB*::Cm^R^	This work
AK1082	*cpxP-lacZ*^*+*^*Y*^*+*^Δ*trxC*::Cm^R^	This work
***E. coli***		
DH5α	F^-^*sup*E44 Δ*lac*U169 (Ф80 *lacZ*ΔM15)*hsd*R17 *rec*A1 *end*A1 *gyr*A96 *thi*-1*rel*A1	[49]
**Plasmids**		
pUC19	rep_pMB1_ Ap^R^	[50]
pUC19-R1	rep_pMB1_ Ap^R^	This work
pWN1	rep_pMB1_ Ap^R^	This work
pKD3	rep_R6Kγ_ Ap^R^ FRT Cm^R^ FRT	[45]
pKD46	rep_pSC101_ts Ap^R^ p_*araBAD*_ γ β exo	[45]
pCP20	rep_pSC101_ts Ap^R^ Cm^R^*cl*857λP_R_*flp*	[51]
pCE37	rep_R6Kγ_ Km^R^ FRT *lacZY* t_*his*_	[44]
pBAD18	rep_pMB1_ Ap^R^ p_*araBAD*_	[52]
pBAD18-*cacA*	rep_pMB1_ Ap^R^ p_*araBAD*_*cacA*	This work
pASK-IBA3plus(pASK)	rep_pMB1_ Ap^R^*tetR* p_*tet*_	IBA
pASK-*cacA*	rep_pMB1_ Ap^R^*tetR* p_*tet*_*cacA*	This work

**Table 2:** Primers used in this study

**Primers**	**Sequence (5’ → 3’)**
Primers for strain and plasmid constructions	
35	GTTGAAATTATTGAGTAGTAGCAACTCACGTTACCAGTAACATATGAATATCCTCCTTAG
36	GACAGGGATGGTGTCTATGGAAAGGAAAACAGGGTTGTTAGTGTAGGCTGGAGCTGCTTC
37	CCCGGCGCAAGAAGGTAAAATGCCTGCTGCGGCAGAATAACATATGAATATCCTCCTTAG
38	TGTCGACAAGACCGGCGGTCTTAAATTATGCGGAAAGTTAGTGTAGGATGGAGCTGCTTC
84	ACATAATCAGGACTCACTGCAGCTTGCGGACGCGCAATAACATATGAATATCCTCCTTAG
85	AATGTCGGCGCTTCTGTTCCCCAGGAAGGCTAATCGTTTAGTGTAGGCTGGAGCTGCTTC
333	TCCTACACTATCGTGTAAAGTAGTTAACATTGAGGATGTACATATGAATATCCTCCTTAG
336	GTTAGCGCGGATACAATAGCGGTATCAGCGACCAGGGTTAGTGTAGGCTGGAGCTGCTTC
337	GGAATTCTAACATTGAGGATGTAATGT
338	ACGCGTCGACTTAAAAATCGCTATATTGCT
367	ACCATGCCACTATTGATTAAAGCCAGTCAGGGGAGAGAACGTGTAGGCTGGAGCTGCTTC
368	GGCCGGTAAAGCAATTTCCGCTCACTCTTCCGTTTGGTCACATATGAATATCCTCCTTAG
393	ATTGCGTGGTCGCGGCTATCTGATGGTTTCCGCTTCATGAGTGTAGGCTGGAGCTGCTTC
394	GATAAAAAATCGGCCTGCATTCGCAGGCCGATGGTTTTTACATATGAATATCCTCCTTAG
451	ACATAATCAGGACTCACTGCAGCTTGCGGACGCGCAATAACTCTAATGCGCTGTTAATCACT
452	GTTGTAAAACGACGGCCAGTGAATCCGTAATCATGGTCATCTAAGCACTTGTCTCCTGTT
453	ACATAATCAGGACTCACTGCAGCTTGCGGACGCGCAATAATTCTCAACGGGGAACATTCC
454	GTTGTAAAACGACGGCCAGTGAATCCGTAATCATGGTCATTACATCCTCAATGTTAACTA
473	TTGCTAGTTCCGTCAAGGGATCACGGGTAGGAGCCACCTTGTGTAGGCTGGAGCTGCTTC
474	GCCAGTCGACAGACTGGCCTTTTTTTGACAAGGGTACTTACATATGAATATCCTCCTTAG
639	GAGGAATAATAAATGTTCGCGCTGGTACTTTTTG
640	TTAAAAATCGCTATATTGCTGCGCAGG
832	ACATAATCAGGACTCACTGCAGCTTGCGGACGCGCAATAACTCAAAAAGAACCCGTCGCC
833	GCAGGGGGGAAATGAGAAAAGGAAGAATAAATAACCCGCCTG
834	CTTTTCTCATTTCCCCCCTGCGCTAACCTCTCGTACACTATCGTGTAAAGTAGTTAACATTGAGGATGTA
835	CTTTTCTCATTTCCCCCCTGCGCTAACCTCACGTACACTATCGTGTAAAGTAGTTAACATTGAGGATGTA
836	CTTTTCTCATTTCCCCCCTGCGCTAACCTCTCCTTCACAATCGTGTAAAGTAGTTAACATTGAGGATGTA
1160	GCTACACCAACACGCCAGGCTTATTCCTGTGGAGTTATATGTGTAGGCTGGAGCTGCTTC
1161	CCATACAGCGCCTTTGTCATTCGACGTATAAAAGGTATTACATATGAATATCCTCCTTAG
1164	ACAATTCTGCTCATTGTCTGCCAACAACTATGGGGATCTCGTGTAGGCTGGAGCTGCTTC
1165	AGTCGCCTTTTTTACTTTTGTTACTGATTTGTAAAAACTACATATGAATATCCTCCTTAG
1166	CGCGTAGCGGGACGTCTTCCGACGTATTCAGAGGTTAGCTGTGTAGGCTGGAGCTGCTTC
1167	GAGGTGAAAACGGGGCACAAGATGCGCCCCGTGGCGTTTACATATGAATATCCTCCTTAG

### β-Galactosidase assay

For data presented in Figures 1C, 1E, 2B, 3C, and 4B, a modified kinetic β-galactosidase assay was performed as previously described [42]. *Salmonella* cultures grown in LB overnight were diluted 50 times in fresh medium and grown for 2 h or 4 h at 37°C. Culture aliquot (80 μl) were added to individual wells of a clear 96-well plate containing 20 μl Reporter Lysis buffer (Promega, Madison, WI, USA). Cells were lysed by freezing at -80°C and subsequent thawing at 37°C for ~20 min. One hundred microliters of 1.32 mg/ml 2-Nitrophenyl β-D-galactopyranoside (ONPG, Sigma-Aldrich, St. Louis, MO, USA) in Z-buffer was then added to each well and mixed thoroughly. OD_415_ for each well was read 20 times within 40 min at 25°C using the Model 680 microplate reader (BioRad, Hercules, CA, USA). β-galactosidase activity is reported in arbitrary units [AU] and represents the rate of ONPG conversion (i.e., Velocity, with units of mOD_415_ per minute) divided by the OD_595_ of the bacterial culture at the time of collection. For data presented in Figures 1A, 1D, and 2A, β-galactosidase assays were performed in triplicate, and the activity in Miller units was determined as described [43]. Data correspond to mean values of two or three independent experiments performed in duplicate.

### Strain construction

Strain AK1052, which encodes a transcriptional fusion of *cpxP-lacZY* on the chromosome, was constructed as described [44]. A Cm^R^ cassette was amplified from pKD3 using the primers 35 and 36 and integrated immediately downstream of the stop codon of the *cpxP* gene on the 14028s chromosome by the one-step gene inactivation method [45]. The junction region of *cpxP* and the Cm^R^ cassette was amplified from the chromosome and confirmed by direct nucleotide sequencing. After removing the Cm^R^ cassette, the *lacZY* transcriptional fusion plasmid pCE37 was integrated into the FLP recombination target sequence immediatel downstream of the *cpxP* gene by FLP-mediated recombination.

Strain AK1053, which encodes a transcriptional fusion of *spy-lacZY* on the chromosome, was constructed as described [44]. A Cm^R^ cassette was amplified from pKD3 using the primers 37 and 38 and integrated immediately downstream of the stop codon of the *spy* gene on the 14028s chromosome by the one-step gene inactivation method [45]. The junction region of *spy* and the Cm^R^ cassette was amplified from the chromosome and confirmed by direct nucleotide sequencing. After removing the Cm^R^ cassette, the *lacZY* transcriptional fusion plasmid pCE37 was integrated into the FLP recombination target sequence immediately downstream of the *spy* gene by FLP-mediated recombination.

Strain AK1054, which encodes a transcriptional fusion of *pgtP-lacZY* on the chromosome, was constructed as described [44]. A Cm^R^ cassette was amplified from pKD3 using the primers 84 and 85 and integrated immediately downstream of the stop codon of the *pgtP* gene on the 14028s chromosome by the one-step gene inactivation method [45]. The junction region of *pgtP* and the Cm^R^ cassette was amplified from the chromosome and confirmed by direct nucleotide sequencing. After removing the Cm^R^ cassette, the *lacZY* transcriptional fusion plasmid pCE37 was integrated into the FLP recombination target sequence immediately downstream of the *pgtP* gene by FLP-mediated recombination.

Strain AK1055, which encodes a transcriptional fusion of *tetA-lacZY* on the chromosome, was constructed by the one-step gene inactivation method [45]. The *tetA* gene was amplified from the MS7953s chromosomal DNA using the primers 451 and 452 and integrated between the *pgtP* gene and the *lacZ* gene in the AK1054 chromosome by the one-step gene inactivation method [45]. Strain AK1056, which harbors a fusion of the *cacA* promoter and *lacZY* genes at the *pgtP* locus, was constructed by a combination of the one-step gene inactivation method and the counterselection method for Tet^s^ colonies. A PCR fragment containing the *cacA* promoter was amplified from *Salmonella* chromosomal DNA using the primers 453 and 454 and recombined into the chromosome, replacing the *tetA* insertion in the strain AK1055. Strain AK1067, which harbors a fusion between the *cacA* promoter and the *lacZY* gene at the *pgtP* locus, was constructed by a combination of the one-step gene inactivation method and the counterselection method for Tet^s^ colonies. A PCR fragment containing the *cacA* promoter was amplified from *Salmonella* chromosomal DNA using the primers 832 and 454 and recombined into the chromosome, replacing the *tetA* insertion in the strain AK1055. Strain AK1068, which harbors *lacZY* genes under the control of a mutant *cacA* promoter with a nucleotide substitution (TC**CTACACT** to TC**GTACACT**) in the -10 region at the *pgtP* locus, was constructed by a combination of the one-step gene inactivation method and the counterselection method for Tet^s^ colonies. A PCR fragment containing the mutant *cacA* promoter was amplified from *Salmonella* chromosomal DNA using the primers 832, 833, 834, and 454 by the asymmetric PCR-based synthesis method [46] and recombined into the chromosome, replacing the *tetA* insertion in the strain AK1055. Strain AK1069, which harbors *lacZY* genes under the control of a mutant *cacA* promoter with two nucleotide substitutions (TC**CTACACT** to AC**GTACACT**) in the -10 region at the *pgtP* locus, was constructed by a combination of the one-step gene inactivation method and the counterselection method for Tet^s^ colonies. A PCR fragment containing the mutant *cacA* promoter was amplified from *Salmonella* chromosomal DNA using the primers 832, 833, 835, and 454 by the asymmetric PCR-based synthesis method [46] and recombined into the chromosome, replacing the *tetA* insertion in the strain AK1055. Strain AK1070, which harbors *lacZY* genes under the control of a mutant *cacA* promoter with two nucleotide substitutions (TC**CTACACT** to TC**CTTCACA**) in the -10 region at the *pgtP* locus, was constructed by a combination of the one-step gene inactivation method and the counterselection method for Tet^s^ colonies. A PCR fragment containing the mutant *cacA* promoter was amplified from *Salmonella* chromosomal DNA using the primers 832, 833, 836, and 454 by the asymmetric PCR-based synthesis method [46] and recombined into the chromosome, replacing the *tetA* insertion in the strain AK1055.

Strain AK1057, which harbors a deletion in the *cpxA* coding region, was constructed by the one-step gene inactivation method [45]. A Cm^R^ cassette was amplified from pKD3 using the primers 393 and 394 and recombined into the 14028s chromosome. Strain AK1058, which harbors a deletion in the *rssB* coding region, was constructed by the one-step gene inactivation method [45]. A Cm^R^ cassette was amplified from pKD3 using the primers 367 and 368 and recombined into the 14028s chromosome. Strain AK1059, which harbors a deletion in the *rpoS* coding region, was constructed by the one-step gene inactivation method [45]. A Cm^R^ cassette was amplified from pKD3 using the primers 473 and 474 and recombined into the 14028s chromosome. Strain AK1060, which harbors a deletion in the *cacA* coding region, was constructed by the one-step gene inactivation method [45]. A Cm^R^ cassette was amplified from pKD3 using the primers 333 and 336 and recombined into the 14028s chromosome. Strain AK1077, which harbors a deletion in the *trxA* coding region, was constructed by the one-step gene inactivation method [45]. A Cm^R^ cassette was amplified from pKD3 using the primers 1160 and 1161 and recombined into the 14028s chromosome. Strain AK1078, which harbors a deletion in the *trxB* coding region, was constructed by the one-step gene inactivation method [45]. A Cm^R^ cassette was amplified from pKD3 using the primers 1164 and 1165 and recombined into the 14028s chromosome. Strain AK1079, which harbors a deletion in the *trxC* coding region, was constructed by the one-step gene inactivation method [45]. A Cm^R^ cassette was amplified from pKD3 using the primers 1166 and 1167 and recombined into the 14028s chromosome.

### Plasmid construction

The pBAD18-*cacA* plasmid, encoding the CacA protein, was constructed by cloning a PCR fragment, generated using the primers 337 and 338 from a pWN1 template, between the *Eco*RI and *Bam*HI sites in the pBAD18plasmid.The pASK-*cacA* plasmid, encoding the CacA protein, was constructed by TA cloning [47] of a PCR fragment, generated using the primers 639 and 640 from a 14028s genomic DNA template, into the pASK-IBA3plus plasmid that had been digested with *Sma*I and T-tailed.

### Screening for a gene that activates the CpxR/CpxA system

Chromosomal DNA prepared from an overnight culture of wild-type strain 14028s was digested with *Sau*3AI (0.01 U/μl) for 4 h. The digested DNA was separated on a 0.8% agarose gel, and 0.5–5 kb fragments were collected and ligated to pUC19 plasmid DNA that had been digested with *Bam*HI and dephosphorylated by alkaline phosphatase. The ligation mixture was transformed into *E. coli* DH5α, and ampicillin-resistant transformants were selected. Plasmid DNA was prepared from a pool of ~100,000 transformants and used to transform the strain AK1052. Transformants were serially diluted and spread onto LB plates containing ampicillin and 40 μg/ml X-gal to obtain 1,000 ~ 10,000 colonies per plate. Plasmids were isolated from colonies that developed a blue color on LB plates containing ampicillin and X-gal. These plasmids were reintroduced into AK1052 by electroporation, and four transformants were selected on LB plates containing ampicillin and X-gal. A random single white colony from the same plate was also selected as a negative control.

## Abbreviations

ATc: Anhydrotetracycline; AU: Arbitrary units; CKO: *Citrobacter koseri*; DDA: *Dickeya dadantii Ech703*; DTT: Dithiothreitol; ECO: *Escherichia coli*; EDTA: Ethylenediaminetetraacetic acid; ENT: *Enterobacter* sp. 638; ESA: *Cronobacter sakazakii*; GST: Glutathione S Transferase; HK: Hisitidine kinase; IPTG: Isopropyl-β-D-thiogalactopyranoside; LB: Luria-Bertani; KPN: *Klebsiella pneumonia*; ONPG: 2-Nitrophenyl β-D-galactopyranoside; ORF: Open reading frame; RAH: *Rahnella sp. Y9602*; RR: Response regulator; SDS: Sodium dodecyl sulfate; STM: *Salmonella enterica* serovar Typhimurium LT2; TCS: Two-component system; TSS: Transcription start site; X-gal: 5-Bromo-4-chloro-3-indolyl-β-D-galactoside.

## Competing interest

The authors declare that they have no competing financial interests.

## Authors’ contributions

AK designed the experiments. AK, HH, WN, HE, KH performed the experiments. AK wrote the manuscript. RU edited the manuscript. All authors read and approved the final manuscript.

## References

[B1] Ulrich LE Zhulin IB The MiST2 database: a comprehensive genomics resource on microbial signal transduction Nucleic Acids Res 2010 38 Database issue D401 407 19900966 10.1093/nar/gkp940 PMC2808908

[B2] Laub MT Goulian M Specificity in two-component signal transduction pathways Annu Rev Genet 2007 41 121 145 10.1146/annurev.genet.41.042007.170548 18076326

[B3] Bijlsma JJ Groisman EA Making informed decisions: regulatory interactions between two-component systems Trends Microbiol 2003 11 8 359 366 10.1016/S0966-842X(03)00176-8 12915093

[B4] Mitrophanov AY Groisman EA Signal integration in bacterial two-component regulatory systems Genes Dev 2008 22 19 2601 2611 10.1101/gad.1700308 18832064 PMC2751022

[B5] Kato A Groisman EA The PhoQ/PhoP regulatory network of *Salmonella enterica* Adv Exp Med Biol 2008 631 7 21 10.1007/978-0-387-78885-2_2 18792679

[B6] Kato A Groisman EA Connecting two-component regulatory systems by a protein that protects a response regulator from dephosphorylation by its cognate sensor Genes Dev 2004 18 18 2302 2313 10.1101/gad.1230804 15371344 PMC517523

[B7] Kox LF Wosten MM Groisman EA A small protein that mediates the activation of a two-component system by another two-component system EMBO J 2000 19 8 1861 1872 10.1093/emboj/19.8.1861 10775270 PMC302009

[B8] Tu X Latifi T Bougdour A Gottesman S Groisman EA The PhoP/PhoQ two-component system stabilizes the alternative sigma factor RpoS in Salmonella enterica Proc Natl Acad Sci USA 2006 103 36 13503 13508 10.1073/pnas.0606026103 16938894 PMC1557385

[B9] Bougdour A Cunning C Baptiste PJ Elliott T Gottesman S Multiple pathways for regulation of sigmaS (RpoS) stability in *Escherichia coli* via the action of multiple anti-adaptors Mol Microbiol 2008 68 2 298 313 10.1111/j.1365-2958.2008.06146.x 18383615

[B10] Eguchi Y Itou J Yamane M Demizu R Yamato F Okada A Mori H Kato A Utsumi R B1500, a small membrane protein, connects the two-component systems EvgS/EvgA and PhoQ/PhoP in *Escherichia coli* Proc Natl Acad Sci USA 2007 104 47 18712 18717 10.1073/pnas.0705768104 17998538 PMC2141842

[B11] Gerken H Charlson ES Cicirelli EM Kenney LJ Misra R MzrA: a novel modulator of the EnvZ/OmpR two-component regulon Mol Microbiol 2009 72 6 1408 1422 10.1111/j.1365-2958.2009.06728.x 19432797 PMC2727453

[B12] Kato A Ohnishi H Yamamoto K Furuta E Tanabe H Utsumi R Transcription of emrKY is regulated by the EvgA-EvgS two-component system in *Escherichia coli* K-12 Biosci Biotechnol Biochem 2000 64 6 1203 1209 10.1271/bbb.64.1203 10923791

[B13] Cosma CL Danese PN Carlson JH Silhavy TJ Snyder WB Mutational activation of the Cpx signal transduction pathway of *Escherichia coli* suppresses the toxicity conferred by certain envelope-associated stresses Mol Microbiol 1995 18 3 491 505 10.1111/j.1365-2958.1995.mmi_18030491.x 8748033

[B14] Kato A Tanabe H Utsumi R Molecular characterization of the PhoP-PhoQ two-component system in *Escherichia coli* K-12: identification of extracellular Mg^2+^-responsive promoters J Bacteriol 1999 181 17 5516 5520 10464230 10.1128/jb.181.17.5516-5520.1999 PMC94065

[B15] Lippa AM Goulian M Feedback inhibition in the PhoQ/PhoP signaling system by a membrane peptide PLoS Genet 2009 5 12 e1000788 10.1371/journal.pgen.1000788 20041203 PMC2789325

[B16] Kato A Chen HD Latify T Groisman EA Reciprocal Control Between a Bacterium's Regulatory System and the Modification Status of its Lipopolysaccharide Mol Cell 2012 47 6 897 908 10.1016/j.molcel.2012.07.017 22921935 PMC3465083

[B17] Vogt SL Raivio TL Just scratching the surface: an expanding view of the Cpx envelope stress response FEMS Microbiol Lett 2012 326 1 2 11 10.1111/j.1574-6968.2011.02406.x 22092948

[B18] Buelow DR Raivio TL Cpx signal transduction is influenced by a conserved N-terminal domain in the novel inhibitor CpxP and the periplasmic protease DegP J Bacteriol 2005 187 19 6622 6630 10.1128/JB.187.19.6622-6630.2005 16166523 PMC1251582

[B19] DiGiuseppe PA Silhavy TJ Signal detection and target gene induction by the CpxRA two-component system J Bacteriol 2003 185 8 2432 2440 10.1128/JB.185.8.2432-2440.2003 12670966 PMC152615

[B20] Isaac DD Pinkner JS Hultgren SJ Silhavy TJ The extracytoplasmic adaptor protein CpxP is degraded with substrate by DegP Proc Natl Acad Sci USA 2005 102 49 17775 17779 10.1073/pnas.0508936102 16303867 PMC1308919

[B21] Snyder WB Davis LJ Danese PN Cosma CL Silhavy TJ Overproduction of NlpE, a new outer membrane lipoprotein, suppresses the toxicity of periplasmic LacZ by activation of the Cpx signal transduction pathway J Bacteriol 1995 177 15 4216 4223 7635808 10.1128/jb.177.15.4216-4223.1995 PMC177165

[B22] Otto K Silhavy TJ Surface sensing and adhesion of *Escherichia coli* controlled by the Cpx-signaling pathway Proc Natl Acad Sci USA 2002 99 4 2287 2292 10.1073/pnas.042521699 11830644 PMC122357

[B23] Raivio TL Laird MW Joly JC Silhavy TJ Tethering of CpxP to the inner membrane prevents spheroplast induction of the Cpx envelope stress response Mol Microbiol 2000 37 5 1186 1197 10.1046/j.1365-2958.2000.02074.x 10972835

[B24] Yamamoto K Ishihama A Characterization of copper-inducible promoters regulated by CpxA/CpxR in *Escherichia coli* Biosci Biotechnol Biochem 2006 70 7 1688 1695 10.1271/bbb.60024 16861804

[B25] McClelland M Sanderson KE Spieth J Clifton SW Latreille P Courtney L Porwollik S Ali J Dante M Du F Complete genome sequence of *Salmonella enterica* serovar Typhimurium LT2 Nature 2001 413 6858 852 856 10.1038/35101614 11677609

[B26] Raivio TL Silhavy TJ The sigmaE and Cpx regulatory pathways:overlapping but distinct envelope stress responses Curr Opin Microbiol 1999 2 2 159 165 10.1016/S1369-5274(99)80028-9 10322173

[B27] Raffa RG Raivio TL A third envelope stress signal transduction pathway in *Escherichia coli* Mol Microbiol 2002 45 6 1599 1611 10.1046/j.1365-2958.2002.03112.x 12354228

[B28] Hagiwara D Sugiura M Oshima T Mori H Aiba H Yamashino T Mizuno T Genome-wide analyses revealing a signaling network of the RcsC-YojN-RcsB phosphorelay system in *Escherichia coli* J Bacteriol 2003 185 19 5735 5746 10.1128/JB.185.19.5735-5746.2003 13129944 PMC193970

[B29] Lee SJ Gralla JD Sigma38 (*rpoS*) RNA polymerase promoter engagement via -10 region nucleotides J Biol Chem 2001 276 32 30064 30071 10.1074/jbc.M102886200 11375988

[B30] Ramachandran VK Shearer N Jacob JJ Sharma CM Thompson A The architecture and ppGpp-dependent expression of the primary transcriptome of *Salmonella Typhimurium* during invasion gene expression BMC Genomics 2012 13 25 10.1186/1471-2164-13-25 22251276 PMC3293720

[B31] Ritz D Beckwith J Roles of thiol-redox pathways in bacteria Annu Rev Microbiol 2001 55 21 48 10.1146/annurev.micro.55.1.21 11544348

[B32] Slamti L Waldor MK Genetic analysis of activation of the Vibrio cholerae Cpx pathway J Bacteriol 2009 191 16 5044 5056 10.1128/JB.00406-09 19542291 PMC2725601

[B33] Stewart EJ Katzen F Beckwith J Six conserved cysteines of the membrane protein DsbD are required for the transfer of electrons from the cytoplasm to the periplasm of *Escherichia coli* EMBO J 1999 18 21 5963 5971 10.1093/emboj/18.21.5963 10545108 PMC1171662

[B34] Hirano Y Hossain MM Takeda K Tokuda H Miki K Structural studies of the Cpx pathway activator NlpE on the outer membrane of *Escherichia coli* Structure 2007 15 8 963 976 10.1016/j.str.2007.06.014 17698001

[B35] Tao K Watanabe S Narita S Tokuda H A periplasmic LolA derivative with a lethal disulfide bond activates the Cpx stress response system J Bacteriol 2010 192 21 5657 5662 10.1128/JB.00821-10 20802033 PMC2953700

[B36] Lippa AM Goulian M Perturbation of the oxidizing environment of the periplasm stimulates the PhoQ/PhoP system in *Escherichia coli* J Bacteriol 2012 194 6 1457 1463 10.1128/JB.06055-11 22267510 PMC3294871

[B37] Kumar JK Tabor S Richardson CC Proteomic analysis of thioredoxin-targeted proteins in *Escherichia coli* Proc Natl Acad Sci USA 2004 101 11 3759 3764 10.1073/pnas.0308701101 15004283 PMC374317

[B38] Hemm MR Paul BJ Miranda-Rios J Zhang A Soltanzad N Storz G Small stress response proteins in *Escherichia coli*: proteins missed by classical proteomic studies J Bacteriol 2010 192 1 46 58 10.1128/JB.00872-09 19734316 PMC2798279

[B39] Kato A Mitrophanov AY Groisman EA A connector of two-component regulatory systems promotes signal amplification and persistence of expression Proc Natl Acad Sci USA 2007 104 29 12063 12068 10.1073/pnas.0704462104 17615238 PMC1924540

[B40] Davis RW Bolstein D Roth JR Advanced bacterial genetics 1980 Cold Spring Harbor Lab, Cold Spring Harbor, N.Y.

[B41] Snavely MD Gravina SA Cheung T-BT Miller CG Maguire ME Magnesium transport in *Salmonella typhimurium*: regulation of *mgtA* and *mgtB* expression J Biol Chem 1991 266 2 824 829 1898738

[B42] Camp AH Losick R A feeding tube model for activation of a cell-specific transcription factor during sporulation in *Bacillus subtilis* Genes Dev 2009 23 8 1014 1024 10.1101/gad.1781709 19390092 PMC2675869

[B43] Miller JH Experiments in molecular genetics 1972 Cold Spring Harbor Laboratory Press, Cold Spring Harbor, NY

[B44] Ellermeier CD Janakiraman A Slauch JM Construction of targeted single copy *lac* fusions using lambda Red and FLP-mediated site-specific recombination in bacteria Gene 2002 290 1–2 153 161 12062810 10.1016/s0378-1119(02)00551-6

[B45] Datsenko KA Wanner BL One-step inactivation of chromosomal genes in *Escherichia coli* K-12 using PCR products Proc Natl Acad Sci USA 2000 97 12 6640 6645 10.1073/pnas.120163297 10829079 PMC18686

[B46] Pan W Ravot E Tolle R Frank R Mosbach R Turbachova I Bujard H Vaccine candidate MSP-1 from *Plasmodium falciparum*: a redesigned 4917 bp polynucleotide enables synthesis and isolation of full-length protein from *Escherichia coli* and mammalian cells Nucleic Acids Res 1999 27 4 1094 1103 10.1093/nar/27.4.1094 9927744 PMC148291

[B47] Zhou MY Gomez-Sanchez CE Universal TA cloning Curr Issues Mol Biol 2000 2 1 1 7 11464915

[B48] Fields PI Groisman EA Heffron F A *Salmonella* locus that controls resistance to microbicidal proteins from phagocytic cells Science 1989 243 4894 Pt 1 1059 1062 2646710 10.1126/science.2646710

[B49] Hanahan D Studies on transformation of *Escherichia coli* with plasmids J Mol Biol 1983 166 4 557 580 10.1016/S0022-2836(83)80284-8 6345791

[B50] Tabor S Richardson CC A bacteriophage T7 RNA polymerase/promoter system for controlled exclusive expression of specific genes Proc Natl Acad Sci USA 1985 82 1074 1078 10.1073/pnas.82.4.1074 3156376 PMC397196

[B51] Cherepanov PP Wackernagel W Gene disruption in *Escherichia coli*: Tc^R^ and Km^R^ cassettes with the option of Flp-catalyzed excision of the antibiotic-resistance determinant Gene 1995 158 1 9 14 10.1016/0378-1119(95)00193-A 7789817

[B52] Guzman L-M Belin D Carson MJ Beckwith J Tight regulation, modulation, and high-level expression by vectors containing the arabinose P_BAD_ promoter J Bacteriol 1995 177 14 4121 4130 7608087 10.1128/jb.177.14.4121-4130.1995 PMC177145

